# Supplementary material for: From acute to persistent infection: revealing phylogenomic variations in Salmonella Agona
Source: PLoS Pathog. 2024 Oct 31;20(10):e1012679. doi: 10.1371/journal.ppat.1012679 (PMC11556752; doi:10.1371/journal.ppat.1012679)
Supplement: S1 Fig — Neighbour-joining phylogeny categorised by year of isolation (inner ring), travel (second ring), most common HC5 clusters (third ring) and carriage status (outer ring) as denoted by colour. (PDF) [file ppat.1012679.s001.pdf]

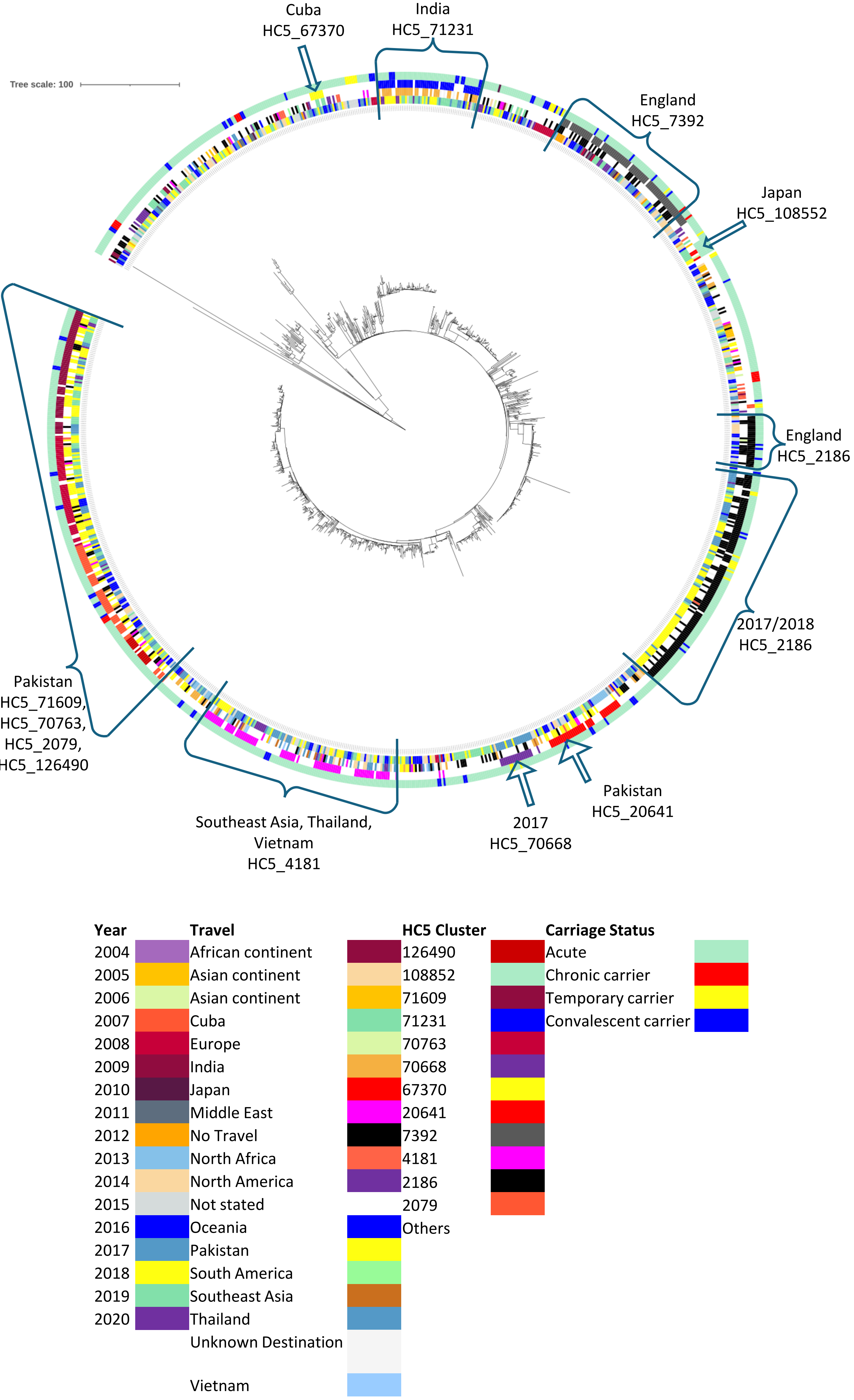
Tree scale: 100
Cuba HC5_67370
India HC5_71231
England HC5_7392
Japan HC5_108552
England HC5_2186
2017/2018 HC5_2186
Pakistan HC5_20641
2017 HC5_70668
Southeast Asia, Thailand, Vietnam HC5_4181
Pakistan HC5_71609, HC5_70763, HC5_2079, HC5_126490
Year
2004
2005
2006
2007
2008
2009
2010
2011
2012
2013
2014
2015
2016
2017
2018
2019
2020
Travel
African continent
Asian continent
Asian continent
Cuba
Europe
India
Japan
Middle East
No Travel
North Africa
North America
Not stated
Oceania
Pakistan
South America
Southeast Asia
Thailand
Unknown Destination
Vietnam
HC5 Cluster
126490
108852
71609
71231
70763
70668
67370
20641
7392
4181
2186
2079
Others
Carriage Status
Acute
Chronic carrier
Temporary carrier
Convalescent carrier
