# Supplementary figures and images for: From acute to persistent infection: revealing phylogenomic variations in Salmonella Agona
Source: PLoS Pathog. 2024 Oct 31;20(10):e1012679. doi: 10.1371/journal.ppat.1012679 (PMC11556752; doi:10.1371/journal.ppat.1012679)

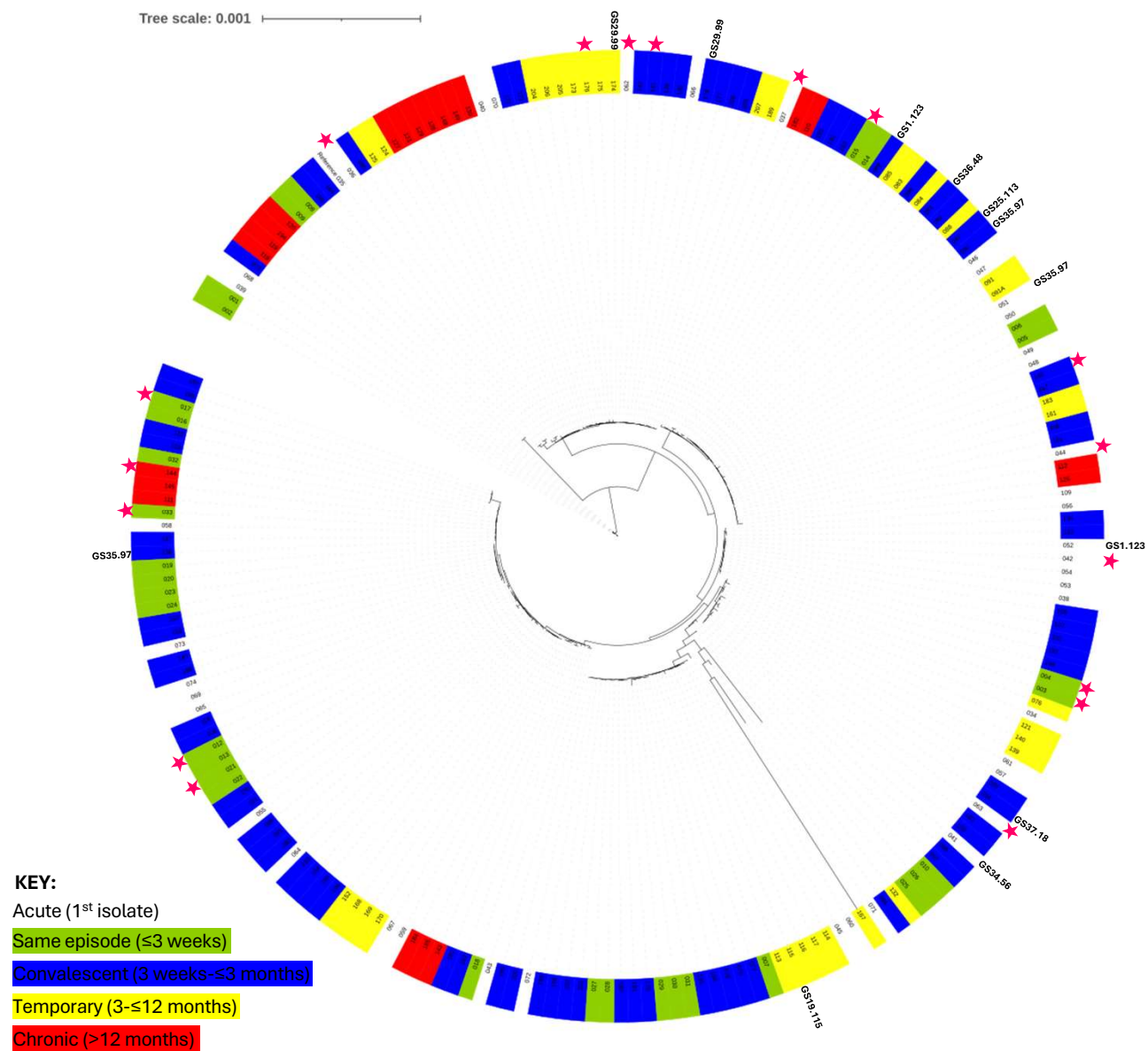

Supplement: S2 Fig — Maximum likelihood tree of hybrid assemblies shows patient carriage status (key), strong biofilm formers (star) & rearranged genomes (bold text) are distributed across the tree. (PDF) [file ppat.1012679.s002.pdf]

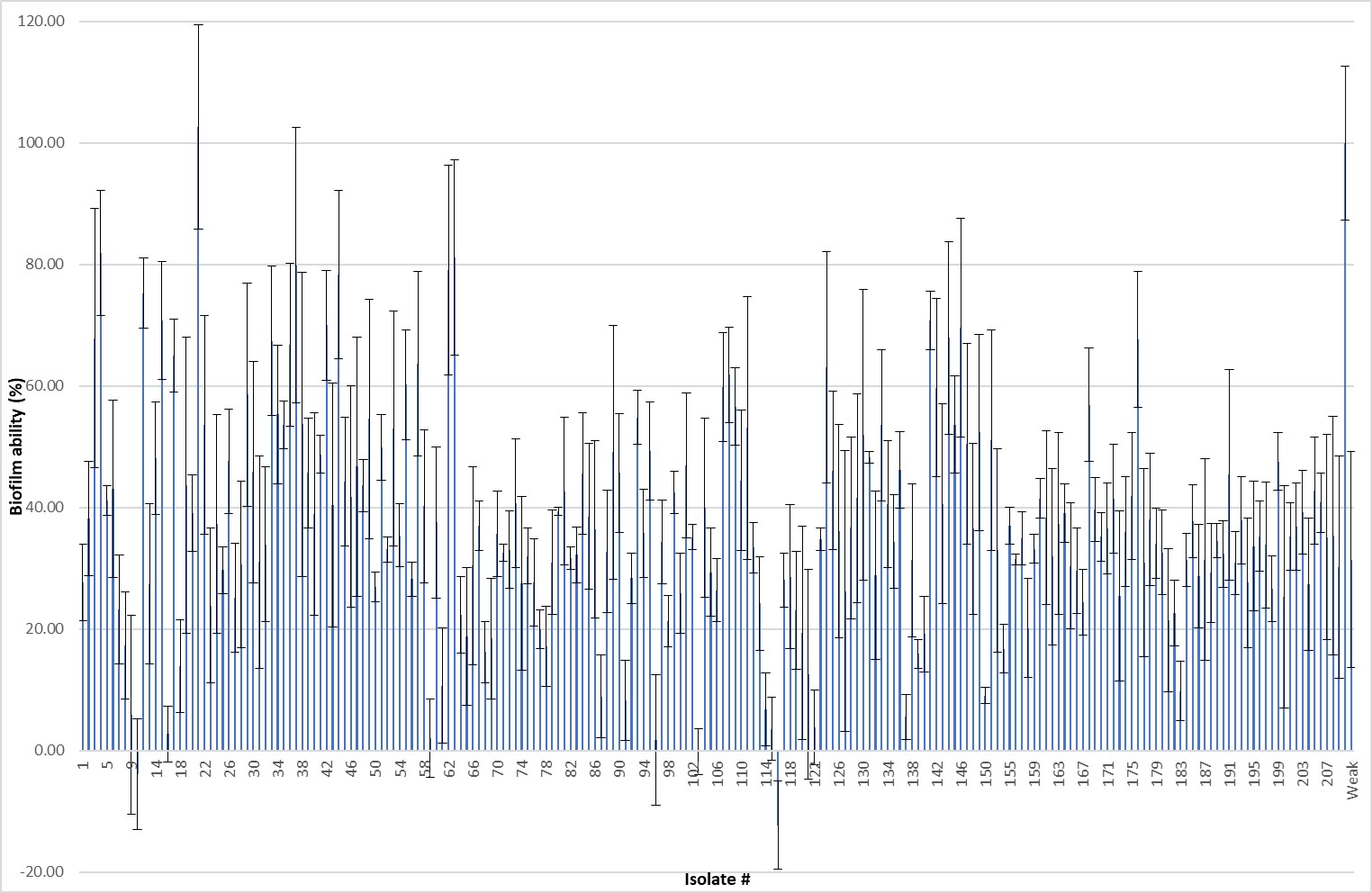

Supplement: S4 Fig — Biofilm ability is given as percentage relative to the control S. Typhimurium strain 14028S (accession: CP001363), which was set at 100% (Prouty and Gunn 2003 [76]; García et al. 2004 [77]; Trampari et al. 2019) [78]. (JPG) [file ppat.1012679.s004.jpg]
