# Supplementary material for: From acute to persistent infection: revealing phylogenomic variations in Salmonella Agona
Source: PLoS Pathog. 2024 Oct 31;20(10):e1012679. doi: 10.1371/journal.ppat.1012679 (PMC11556752; doi:10.1371/journal.ppat.1012679)
Supplement: S3 Fig — For P6, carbon source utilisation was also assessed for another two intermediate isolates. Keys included with each graph showing isolates in chronical order from the original at the top to last. Biolog PM1 panel carbon sources are alphabetically ordered left to right, top to bottom. (PDF) [file ppat.1012679.s003.pdf]

Patient 1

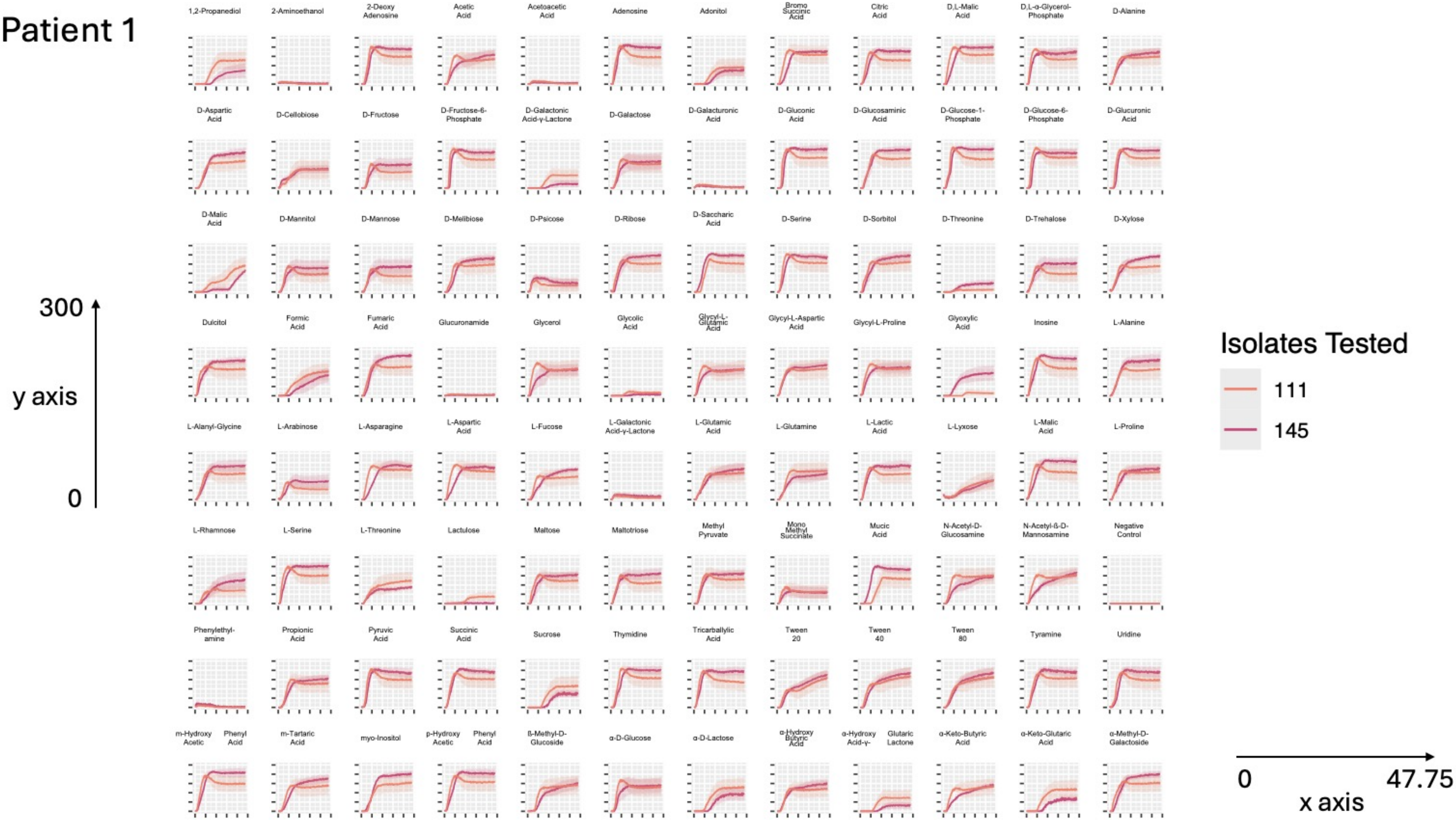
300
y axis
0
1,2-Propanediol
2-Aminoethanol
2-Deoxy Adenosine
Acetic Acid
Acetoacetic Acid
Adenosine
Adonitol
Bromo Succinic Acid
Citric Acid
D,L-Malic Acid
D,L-α-Glycerol-Phosphate
D-Alanine
D-Aspartic Acid
D-Cellobiose
D-Fructose
D-Fructose-6-Phosphate
D-Galactonic Acid-γ-Lactone
D-Galactose
D-Galacturonic Acid
D-Gluconic Acid
D-Glucosaminic Acid
D-Glucose-1-Phosphate
D-Glucose-6-Phosphate
D-Glucuronic Acid
D-Malic Acid
D-Mannitol
D-Mannose
D-Melibiose
D-Psicose
D-Ribose
D-Saccharic Acid
D-Serine
D-Sorbitol
D-Threonine
D-Trehalose
D-Xylose
Dulcitol
Formic Acid
Fumaric Acid
Glucuronamide
Glycerol
Glycolic Acid
Glycyl-L-Glutamic Acid
Glycyl-L-Aspartic Acid
Glycyl-L-Proline
Glyoxylic Acid
Inosine
L-Alanine
L-Alanyl-Glycine
L-Arabinose
L-Asparagine
L-Aspartic Acid
L-Fucose
L-Galactonic Acid-γ-Lactone
L-Glutamic Acid
L-Glutamine
L-Lactic Acid
L-Lyxose
L-Malic Acid
L-Proline
L-Rhamnose
L-Serine
L-Threonine
Lactulose
Maltose
Maltotriose
Methyl Pyruvate
Mono Methyl Succinate
Mucic Acid
N-Acetyl-D-Glucosamine
N-Acetyl-ß-D-Mannosamine
Negative Control
Phenylethyl-amine
Propionic Acid
Pyruvic Acid
Succinic Acid
Sucrose
Thymidine
Tricarballylic Acid
Tween 20
Tween 40
Tween 80
Tyramine
Uridine
m-Hydroxy Phenyl Acetic Acid
m-Tartaric Acid
myo-Inositol
p-Hydroxy Phenyl Acetic Acid
ß-Methyl-D-Glucoside
α-D-Glucose
α-D-Lactose
α-Hydroxy Butyric Acid
α-Hydroxy Glutaric Acid-γ-Lactone
α-Keto-Butyric Acid
α-Keto-Glutaric Acid
α-Methyl-D-Galactoside
Isolates Tested
111
145
0
47.75
x axis

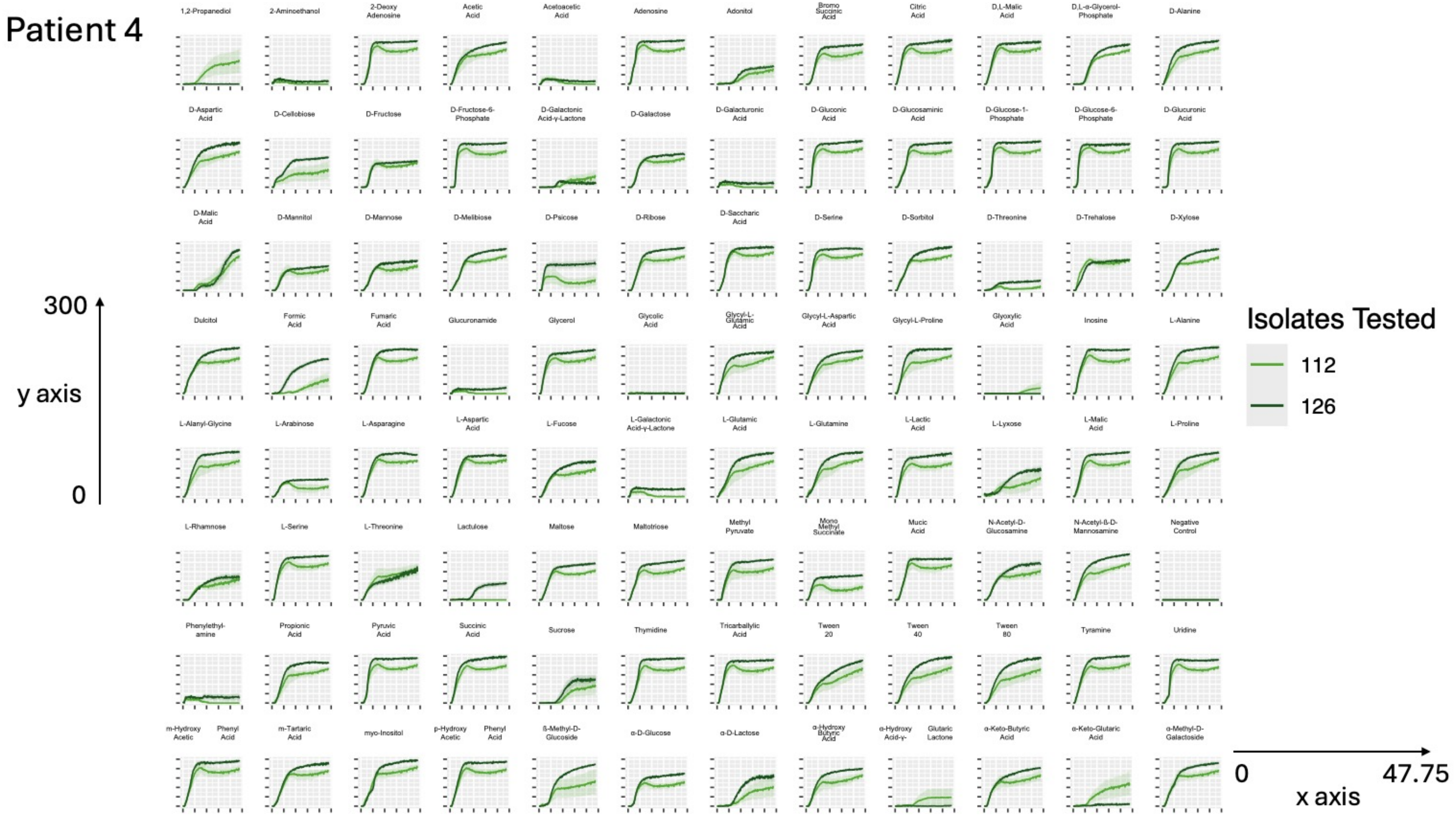

Patient 4
1,2-Propanediol
2-Aminoethanol
2-Deoxy Adenosine
Acetic Acid
Acetoacetic Acid
Adenosine
Adonitol
Bromo Succinic Acid
Citric Acid
D,L-Malic Acid
D,L-α-Glycerol-Phosphate
D-Alanine
D-Aspartic Acid
D-Cellobiose
D-Fructose
D-Fructose-6-Phosphate
D-Galactonic Acid-γ-Lactone
D-Galactose
D-Galacturonic Acid
D-Gluconic Acid
D-Glucosaminic Acid
D-Glucose-1-Phosphate
D-Glucose-6-Phosphate
D-Glucuronic Acid
D-Malic Acid
D-Mannitol
D-Mannose
D-Melibiose
D-Psicose
D-Ribose
D-Saccharic Acid
D-Serine
D-Sorbitol
D-Threonine
D-Trehalose
D-Xylose
Dulcitol
Formic Acid
Fumaric Acid
Glucuronamide
Glycerol
Glycolic Acid
Glycyl-L-Glutamic Acid
Glycyl-L-Aspartic Acid
Glycyl-L-Proline
Glyoxylic Acid
Inosine
L-Alanine
L-Alanyl-Glycine
L-Arabinose
L-Asparagine
L-Aspartic Acid
L-Fucose
L-Galactonic Acid-γ-Lactone
L-Glutamic Acid
L-Glutamine
L-Lactic Acid
L-Lyxose
L-Malic Acid
L-Proline
L-Rhamnose
L-Serine
L-Threonine
Lactulose
Maltose
Maltotriose
Methyl Pyruvate
Mono Methyl Succinate
Mucic Acid
N-Acetyl-D-Glucosamine
N-Acetyl-ß-D-Mannosamine
Negative Control
Phenylethyl-amine
Propionic Acid
Pyruvic Acid
Succinic Acid
Sucrose
Thymidine
Tricarballylic Acid
Tween 20
Tween 40
Tween 80
Tyramine
Uridine
m-Hydroxy Phenyl Acetic Acid
m-Tartaric Acid
myo-Inositol
p-Hydroxy Phenyl Acetic Acid
ß-Methyl-D-Glucoside
α-D-Glucose
α-D-Lactose
α-Hydroxy Butyric Acid
α-Hydroxy Glutaric Acid-γ-Lactone
α-Keto-Butyric Acid
α-Keto-Glutaric Acid
α-Methyl-D-Galactoside
300
y axis
0
Isolates Tested
112
126
0
47.75
x axis

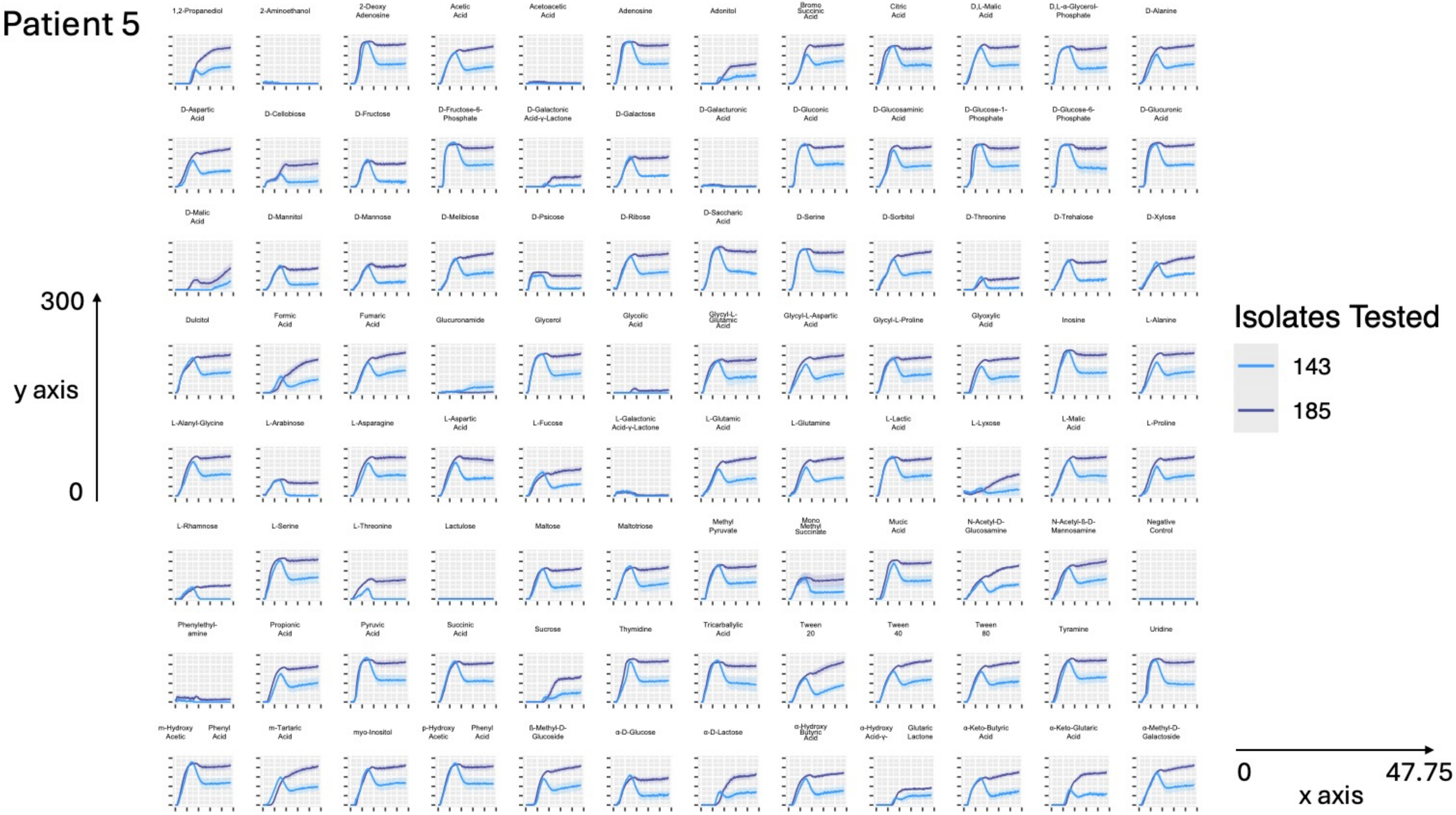
Patient 5
1,2-Propanediol
2-Aminoethanol
2-Deoxy Adenosine
Acetic Acid
Acetoacetic Acid
Adenosine
Adonitol
Bromo Succinic Acid
Citric Acid
D,L-Malic Acid
D,L-α-Glycerol-Phosphate
D-Alanine
D-Aspartic Acid
D-Cellobiose
D-Fructose
D-Fructose-6-Phosphate
D-Galactonic Acid-γ-Lactone
D-Galactose
D-Galacturonic Acid
D-Gluconic Acid
D-Glucosaminic Acid
D-Glucose-1-Phosphate
D-Glucose-6-Phosphate
D-Glucuronic Acid
D-Malic Acid
D-Mannitol
D-Mannose
D-Melibiose
D-Psicose
D-Ribose
D-Saccharic Acid
D-Serine
D-Sorbitol
D-Threonine
D-Trehalose
D-Xylose
Dulcitol
Formic Acid
Fumaric Acid
Glucuronamide
Glycerol
Glycolic Acid
Glycyl-L-Glutamic Acid
Glycyl-L-Aspartic Acid
Glycyl-L-Proline
Glyoxylic Acid
Inosine
L-Alanine
L-Alanyl-Glycine
L-Arabinose
L-Asparagine
L-Aspartic Acid
L-Fucose
L-Galactonic Acid-γ-Lactone
L-Glutamic Acid
L-Glutamine
L-Lactic Acid
L-Lyxose
L-Malic Acid
L-Proline
L-Rhamnose
L-Serine
L-Threonine
Lactulose
Maltose
Maltotriose
Methyl Pyruvate
Mono Methyl Succinate
Mucic Acid
N-Acetyl-D-Glucosamine
N-Acetyl-ß-D-Mannosamine
Negative Control
Phenylethyl-amine
Propionic Acid
Pyruvic Acid
Succinic Acid
Sucrose
Thymidine
Tricarballylic Acid
Tween 20
Tween 40
Tween 80
Tyramine
Uridine
m-Hydroxy Phenyl Acetic Acid
m-Tartaric Acid
myo-Inositol
p-Hydroxy Phenyl Acetic Acid
ß-Methyl-D-Glucoside
α-D-Glucose
α-D-Lactose
α-Hydroxy Butyric Acid
α-Hydroxy Glutaric Acid-γ-Lactone
α-Keto-Butyric Acid
α-Keto-Glutaric Acid
α-Methyl-D-Galactoside
300
y axis
0
Isolates Tested
143
185
0
x axis
47.75

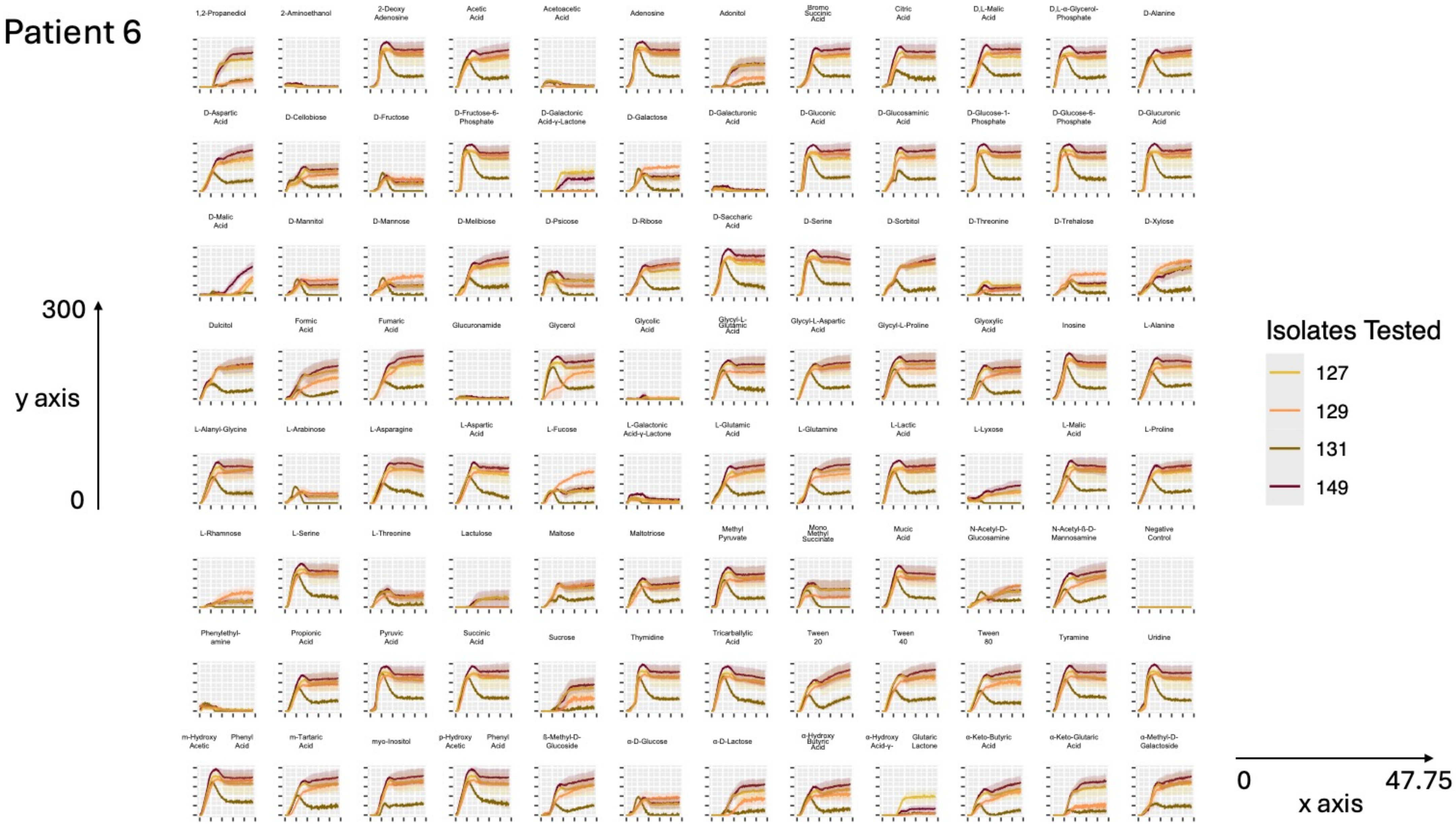
Patient 6
1,2-Propanediol
2-Aminoethanol
2-Deoxy Adenosine
Acetic Acid
Acetoacetic Acid
Adenosine
Adonitol
Bromo Succinic Acid
Citric Acid
D,L-Malic Acid
D,L-α-Glycerol-Phosphate
D-Alanine
D-Aspartic Acid
D-Cellobiose
D-Fructose
D-Fructose-6-Phosphate
D-Galactonic Acid-γ-Lactone
D-Galactose
D-Galacturonic Acid
D-Gluconic Acid
D-Glucosaminic Acid
D-Glucose-1-Phosphate
D-Glucose-6-Phosphate
D-Glucuronic Acid
D-Malic Acid
D-Mannitol
D-Mannose
D-Melibiose
D-Psicose
D-Ribose
D-Saccharic Acid
D-Serine
D-Sorbitol
D-Threonine
D-Trehalose
D-Xylose
Dulcitol
Formic Acid
Fumaric Acid
Glucuronamide
Glycerol
Glycolic Acid
Glycyl-L-Glutamic Acid
Glycyl-L-Aspartic Acid
Glycyl-L-Proline
Glyoxylic Acid
Inosine
L-Alanine
L-Alanyl-Glycine
L-Arabinose
L-Asparagine
L-Aspartic Acid
L-Fucose
L-Galactonic Acid-γ-Lactone
L-Glutamic Acid
L-Glutamine
L-Lactic Acid
L-Lyxose
L-Malic Acid
L-Proline
L-Rhamnose
L-Serine
L-Threonine
Lactulose
Maltose
Maltotriose
Methyl Pyruvate
Mono Methyl Succinate
Mucic Acid
N-Acetyl-D-Glucosamine
N-Acetyl-ß-D-Mannosamine
Negative Control
Phenylethyl-amine
Propionic Acid
Pyruvic Acid
Succinic Acid
Sucrose
Thymidine
Tricarballylic Acid
Tween 20
Tween 40
Tween 80
Tyramine
Uridine
m-Hydroxy Phenyl Acetic Acid
m-Tartaric Acid
myo-Inositol
p-Hydroxy Phenyl Acetic Acid
ß-Methyl-D-Glucoside
α-D-Glucose
α-D-Lactose
α-Hydroxy Butyric Acid
α-Hydroxy Glutaric Acid-γ-Lactone
α-Keto-Butyric Acid
α-Keto-Glutaric Acid
α-Methyl-D-Galactoside
300
y axis
0
Isolates Tested
127
129
131
149
0
47.75
x axis
