# Supplementary material for: From acute to persistent infection: revealing phylogenomic variations in Salmonella Agona
Source: PLoS Pathog. 2024 Oct 31;20(10):e1012679. doi: 10.1371/journal.ppat.1012679 (PMC11556752; doi:10.1371/journal.ppat.1012679)
Supplement: S1 Data — (PDF) [file ppat.1012679.s010.pdf]

Supporting Methods

For each sample, 250 ng of HMW genomic DNA was concentrated into a volume of 6.25 μL using 1X AMPure beads (A63881, Beckman Coulter), following the manufacturer's instructions, before being end-prepped using 0.875 μL and 0.375 μL Ultra II end-prep reaction buffer and enzyme mix, respectively (E7646, New England Biosciences (NEB)) by heating at 20 °C for 5 mins and 65 °C for 5 mins. End-prepped DNA (3.75 μL) were individually barcoded using 1.25 μL of native barcode (one unique barcode for each sample), 1 μL Blunt/TA ligase master mix (M0367, NEB) and 4 μL 5X NEBNext quick ligation reaction buffer (B6058, NEB), by heating at 20 °C for 2 hours and 65 °C for 10 mins. 48 barcoded DNA samples were then pooled before being bead-cleaned using 0.6X AMPure beads with two washes of 700 μL short fragments buffer and one wash of 100 μL of 70 % ethanol before DNA was eluted in 35 μL of nuclease-free water. Sequencing adapters were ligated to 30 μL of this clean library using 5 μL Adapter Mix II alongside 5 μL T4 DNA quick ligase (E6056, NEB) and 10 μL quick ligation buffer (B6058, NEB) by incubating at room temperature for 20 mins. Adaptor-ligated DNA was bead-cleaned using a 0.6X AMPure beads with two washes of 125 μL short fragments buffer before DNA was eluted in 15 μL of elution buffer. The DNA from the last step (200–400 ng) was loaded onto an ONT MinION sequencing flow cell as directed by the manufacturer.
